# Simulated global change: contrasting short and medium term growth and reproductive responses of a common alpine/Arctic cushion plant to experimental warming and nutrient enhancement

**DOI:** 10.1186/2193-1801-3-157

**Published:** 2014-03-22

**Authors:** Juha M Alatalo, Chelsea J Little

**Affiliations:** Department of Ecology and Genetics, Uppsala University, Campus Gotland, SE-621 67 Visby, Sweden

**Keywords:** Alpine, Biomass, Climate change, Cover, Fruit production, Meadow, *Silene acaulis*, Tundra

## Abstract

Cushion plants are important components of alpine and Arctic plant communities around the world. They fulfill important roles as facilitators, nurse plants and foundation species across trophic levels for vascular plants, arthropods and soil microorganisms, the importance of these functions increasing with the relative severity of the environment. Here we report results from one of the few experimental studies simulating global change impacts on cushion plants; a factorial experiment with warming and nutrient enhancement that was applied to an alpine population of the common nurse plant, *Silene acaulis*, in sub-arctic Sweden. Experimental perturbations had significant short-term impacts on both stem elongation and leaf length. *S. acaulis* responded quickly by increasing stem elongation and (to a lesser extent) leaf length in the warming, nutrient, and the combined warming and nutrient enhancements. Cover and biomass also initially increased in response to the perturbations. However, after the initial positive short-term responses, *S. acaulis* cover declined in the manipulations, with the nutrient and combined warming and nutrient treatments having largest negative impact. No clear patterns were found for fruit production. Our results show that *S. acaulis* living in harsh environments has potential to react quickly when experiencing years with favorable conditions, and is more responsive to nutrient enhancement than to warming in terms of vegetative growth. While these conditions have an initial positive impact, populations experiencing longer-term increased nutrient levels will likely be negatively affected.

## Introduction

Polar and alpine ecosystems are assumed to be particularly vulnerable to climate change as their organisms dwell at temperatures just above the zero degree threshold for a very short summer growing season. Predicted changes in climate over the next 100 years are expected to be substantial in arctic and sub-arctic regions (IPCC 2007), with the potential to perturb these temperature and freezing patterns. While warming is often the focus of climate change projections, Arctic and alpine areas are also affected by other types of global changes. For instance, atmospheric nitrogen input has almost doubled in the arctic during the last century due to acidic depositions (Neftel et al. 1985), and further increase is anticipated because of predicted changes in climate (Van Cleve et al. 1990). Nutrient availability is also often a limiting factor for tundra plant growth, with nitrogen (N) and phosphorus (P) as the key limiting elements (Shaver and Kummerow 1992).

Due to predictions of increasing warming and nutrient deposition, a number of experiments have addressed the potential impact of environmental change in Arctic and alpine areas on singular species of vascular plants (Klanderud 2008), bryophytes (Molau and Alatalo 1998; Jägerbrand et al. 2009), lichens (Alatalo 1998; Cornelissen et al. 2001; Jägerbrand et al. 2009), functional groups (Dormann and Woodin 2002), whole plant communities (Alatalo 1998), bacteria (Rinnan et al. 2009), fungi (Olsrud et al. 2010), and arthropods (Bokhorst et al. 2008; Hågvar and Klanderud 2009; Makkonen et al. 2011). Most experimental global change studies in the Arctic have focused on vascular plants (e.g. Arft et al. 1999; Dorji et al. 2013), especially on graminoids and dwarf shrubs, which are commonly the dominant life forms of plants in the alpine and Arctic regions. Factorial experiments combining temperature and nutrient manipulations have found mixed effects, with abundances sometimes increasing and sometimes decreasing at both the species (Klanderud 2008) and functional group (Graglia et al. 2001) levels. Effects have also shifted between the first year of manipulations and longer-term impacts as experiments continued (Arft et al. 1999; Robinson et al. 1998).

Amidst this uncertainty, cushion plants have not been given the same attention even though they make up a major part of some alpine and Arctic vegetation communities (but see Stenström et al. 1997; Alatalo and Totland 1997). Cushion plants are distributed globally in harsh polar and alpine regions, where they are of great importance as they often function as facilitators, nurse plants and foundation species in the severe environments and have a positive effect on other species across trophic levels (Cavieres and Arroyo 2002; Arroyo and Cavieres 2003; Molenda et al. 2012; Reid and Lortie 2012; Roy et al. 2013). The number of studies on cushion plants has increased rapidly during the last decade (Reid et al. 2010) and cushion plants have been shown to inhibit loss of phylogenetic diversity in severe environments, where they can act as “micro-refugia” to less stress tolerant species (Butterfield et al. 2013). Similarly, cushion plants have been shown to enhance species richness more in systems with lower local diversity through their role as nurse plants, sustaining diversity under harsh conditions (Cavieres et al. 2014). For instance, Antonsson et al. (2009) found that the cushion plant *Silene acaulis* had a significant nurse plant effect above a certain elevation threshold in alpine sub-arctic Sweden, indicating that the facilitation is increasingly important at higher elevations with more severe environments. Likewise, Yang et al. (2010) found that positive associations between the cushion plant *Arenaria polytrichoides* and other plants increased with elevation in the Sino-Himalayas. *S. acaulis* has also been shown to function as facilitator for arthropods (Molina-Montenegro et al. 2006; Molenda et al. 2012).

As predicted global change lessens the environmental severity of these high-elevation and high-latitude habitats, cushion plants may still play an essential role. A combined removal and temperature enhancement experiment showed that removing *Azorella madreporica* cushion plants that acted as nurse plants decreased survival, biomass, and photochemical efficiency of the grass *Hordeum comosum* in an alpine site in Los Andes, Chile. In the same study seedling survival was enhanced by cushions, even under warmer conditions, demonstrating their importance as facilitators even under potentially warmer conditions (Cavieres and Sierra-Almeida 2012). However, the study gave no information regarding the impact of the temperature enhancement on the growth, phenology or reproductive performance of the nurse plant *A. madreporica*.

The fact that there have been few experimental studies (to our knowledge only Stenström et al. 1997; Alatalo and Totland 1997; Robinson et al. 1998; Le Roux et al. 2005; Day et al. 2009) of the impact of global change on cushion plants themselves represents a significant gap in knowledge, considering their important function as facilitator and nurse plants in alpine, polar and other harsh environments. The rarity of experimental studies on climate change is evidenced by the fact that a recent review of the ecological literature on cushion plants (Reid et al. 2010) did not acknowledge the existing experimental studies. Instead, information has been gleaned primarily from observational studies, which suggest that the longevity of plants may buffer them against changing climate variability (Morris et al. 2008) and that these long-lived cushion plants may be less sensitive to increased climate variability than short-lived plants. Furthermore, none of the existing studies that we are aware of address global change variables other than temperature.

The few existing experimental warming studies have shown contrasting vegetative growth responses to the perturbations, ranging from negative impact (Le Roux et al. 2005) and conservative responses (Stenström et al. 1997) to positive responses (Day et al. 2009). While lacking an experimental approach, a monitoring study on *Diapensia lapponica* revealed that the cushion plants were sensitive to earlier snowmelt which caused increased mortality on wind-exposed ridges that had the thinnest snow cover and early melt off of snow; during the five-year period with three years of unusually early snowmelt, the live biomass and the flowering of *D. lapponica* declined by 22% and 55%, respectively (Molau 1996). An experimental study with temperature enhancement by Open Top Chambers (OTCs) on *Saxifraga oppositifolia* at three contrasting sites (Swiss Alps, subarctic Sweden and High Arctic Canada) found little influence on phenology and growth from the perturbations (Stenström et al. 1997). Robinson et al. (1998) found no effect of temperature enhancement on the cover of *S. oppositifolia* in high Arctic Svalbard, but nutrient addition caused a significant decrease in over a five year period, the cover decreasing over the whole period. However, Alatalo and Totland (1997) found in their temperature enhancement study that cushions of *S. acaulis* inside the OTCs started flowering substantially earlier than control cushions experiencing ambient temperature. Both the male and female phases developed faster in the OTCs and capsules (fruits) matured earlier, and the cushions produced more mature seeds and had a higher seed/ovule ratio contributing to an overall positive response in reproductive terms (Alatalo and Totland 1997).

Thus, our understanding of whether alpine cushion plants respond to global change in the same way as other vascular plants is to date incomplete and inconclusive. In the present study we examine the potential impact of a factorial experiment with temperature and nutrient enhancement on vegetative growth and number of fruits of *S. acaulis.* We address the following questions: (1) Are there differences in growth response of *S. acaulis* experiencing different perturbations (increased temperature, nutrient addition, and combined temperature enhancement and nutrient addition); 2) Do growth and reproductive responses differ from one another; and (3) Do the responses differ among years.

## Results

### Effect on Stem elongation

**Figure 1:**
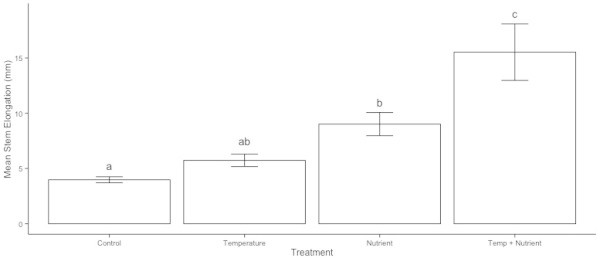
***S. acaulis*** **stem elongation after three years of simulated global change.** Response of *S. acaulis* plants by treatment (control, temperature enhancement, nutrient enhancement and combined temperature and nutrient enhancement) after three years of simulated global change. Error bars represent one standard error and letters denote groupings from posthoc testing, with different letters indicating significant differences (p < 0.05).

A three-factor model of year, warming, and nutrient treatments explained significant variation in stem elongation in this experiment (F = 14.41, d.f. = 7,52, p < 0.001). The responses were consistent across years, with both warming and nutrient manipulation having significant effects (Table 1). Both the warming and nutrient treatments, as well as the combined treatment, increased stem elongation (Figure 1). In all three years, the largest differences were between control plants and the combined nutrient and warming treatment. The nutrient enhancement also had a stronger effect than the warming: there were larger differences in response between control and nutrient plants than between control and warming plants, and the effect of the combined nutrient-warming treatment was stronger than that of the warming treatment alone.

**Table 1:** **Model results for** ***S. acaulis*** **response to simulated global change**

***a)*** **Stem elongation**				
Factor effects	df	MS	F value	p
Temperature	1	4.227	27.496	<0.0001
Nutrient	1	9.731	63.298	<0.0001
Year	1	0.956	6.221	0.02
Nutrient x Year	1	0.093	0.611	0.44
Temperature x Year	1	0.200	1.306	0.26
Nutrient x Temperature	1	0.030	0.196	0.66
Temp x Nutrient x Year	1	0.266	1.732	0.19
Residual	52	0.153		
***b*** **) Leaf length**				
Factor effects	df	MS	F value	p
Temperature	1	0.242	11.598	0.001
Nutrient	1	0.357	17.076	< 0.001
Year	1	0.204	9.783	0.003
Nutrient x Year	1	0.006	0.263	0.61
Temperature x Year	1	< 0.001	0.005	0.94
Nutrient x Temperature	1	< 0.001	0.002	0.97
Temp x Nutrient x Year	1	0.009	0.447	0.51
Residual	52	0.021		
***c*** **) Cover**				
Factor effects	df	MS	F value	p
Temperature	1	197.928	8.849	0.003
Nutrient	1	33.660	1.505	0.22
Year	1	23.100	1.033	0.31
Nutrient x Year	1	183.143	8.188	0.005
Temperature x Year	1	10.968	0.490	0.49
Nutrient x Temperature	1	59.083	2.642	0.10
Temp x Nutrient x Year	1	17.594	0.787	0.38
Residual	88	22.368		
***d*** **) Fruit production**				
Factor effects	df	MS	F value	p
Temperature	1	15.782	10.096	0.002
Nutrient	1	0.601	0.385	0.54
Year	1	9.791	6.264	< 0.001
Nutrient x Year	1	6.356	4.066	0.002
Temperature x Year	1	1.762	1.127	0.35
Nutrient x Temperature	1	5.161	3.302	0.07
Temp x Nutrient x Year	1	1.460	0.934	0.46
Residual	96	1.563		

Analyses of variance for a) stem elongation (log transformed) 1996–1998, b) leaf length 1996–1998, c) cover 1995–2001, and (d) fruit production (log transformed) 1995–2000 of *Silene acaulis*. The factor and interaction effects are from a three factor ANOVA with Temperature, Nutrient and Year as fixed factors.

### Effect on Leaf length and leaf width

The ANOVA model of year, warming, and nutrient treatments fit the variation in leaf length (F = 5.59, d.f. = 7,52, p < 0.001), but not leaf width (F = 1.51, d.f. = 7,52, p > 0.10). This may have been because of marginally significant differences in leaf width by year alone (F = 3.47, d.f. = 1,58, p = 0.067).

**Figure 2:**
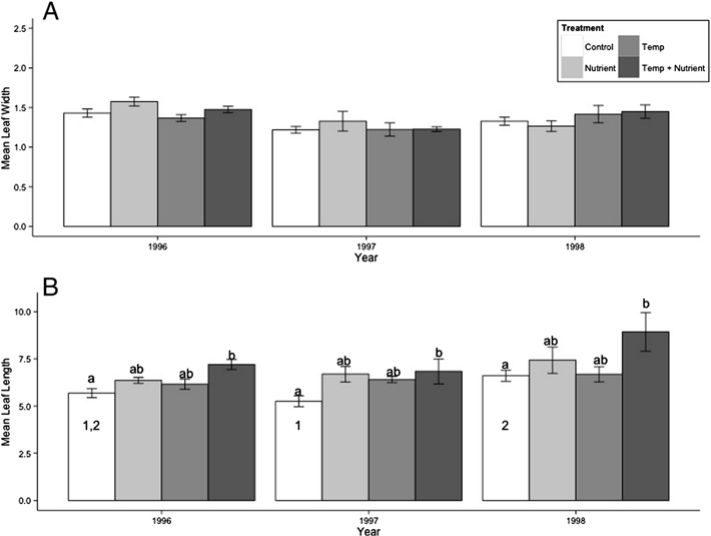
**Response to simulated global change in** ***S. acaulis*** **leaf size.** Leaf width **(A)** and length **(B)** of *S. acaulis* plants by treatment and year. Letters represent groupings of treatments from post-hoc testing within each year only, with different letters indicating significant differences (p < 0.05). Numbers within the bars for control measurements represent groupings of years from post-hoc testing of this treatment type.

Like stem elongation, leaf length also increased when experiencing warming and nutrient enhancement, while year also had a significant effect (Table 1). However, responses were not as strong as for stem elongation (Figure 2). The main effect came from the combined nutrient and warming treatment. For each single treatment alone, there were few differences from the leaf length of plants in the control plots. Only in 1997 did plants from the nutrient treatment have significantly longer leaves than those in the control treatment, and there was never a significant difference between the control and warming treatments (Figure 2).

### Effect on cover and biomass of Silene acaulis

**Figure 3:**
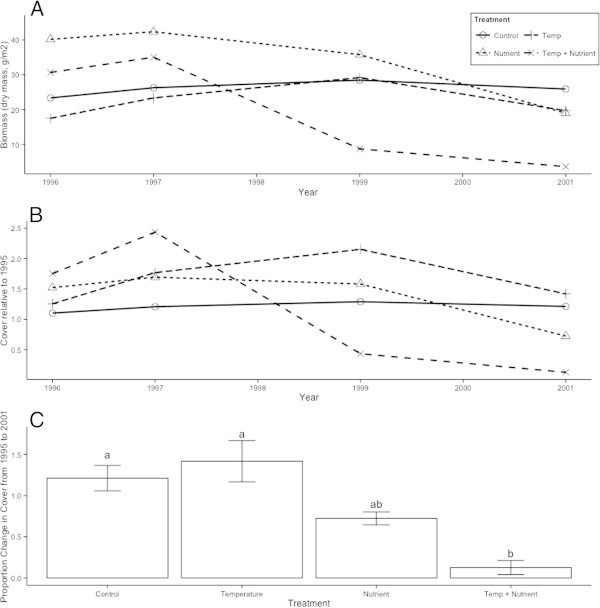
***S. acaulis*** **biomass and cover response to simulated global change. (A)** Dry biomass of *S. acaulis* in simulated global change treatments from 1996 through 2001. **(B)** Changes in *S. acaulis* cover relative to 1995, the first year of the study; for all years except 2001, there were no significant differences between treatments. **(C)** Proportion of original cover extent at the end of the study in 2001, with different letters indicating significant differences between treatments in posthoc testing (p < 0.05).

For cover, the ANOVA model of year, nutrient, and temperature manipulations significantly explained variation (F = 4.105, d.f. = 7, 68, p < 0.001). While year was not a significant main effect, there were trends of growing and shrinking cushion size in different years, best visualized by the biomass metric, which was a direct linear conversion of cover (Molau 2010). For instance, in 1995 before the nutrient treatments were applied, *S. acaulis* plants had a mean biomass of 21.09 g/m^2^ (n = 20, s.d. = 9.96) with a non-significant trend towards smaller plants in the warming treatments, but by 1999, the biomass across treatments had risen to 26.78 g/m^2^ (n = 20, s.d. = 17.38; Figure 3). However, in 2001 it had dropped to 18.82 g/m^2^ (n = 20, s.d. = 10.90). These changes were partially driven by factors unrelated to the treatments, as they matched the trajectories of biomass in the control plots, which had increased by 25 percent by 1999 but then dropped back to 16 percent of its original value by 2001 (Figure 3). Posthoc testing showed that from 1995 through 1999, there were no differences between treatments in each individual year.

Over the course of the entire study period, however, the treatments did have significant effects (Table 1). The temperature manipulation consistently decreased cover. The nutrient manipulation interacted significantly with both year and temperature to determine cover response (Table 1), resulting in different changes in cover depending on the year and whether the nutrient amendment was combined with warming. In both the nutrient and the combined temperature and nutrient treatment, after seven years of warming mean cover in 2001 was less than it had been in 1995, whereas in the temperature treatment it had grown slightly (Figure 3). However, in both the nutrient-only and temperature-only treatment, *S. acaulis* initially responded by increasing in size, before the responses eventually differentiated in their direction. Only in the combined nutrient and temperature treatment did cover decrease in its short-term response by 1999.

### Effect on fruit production of Silene acaulis

**Figure 4:**
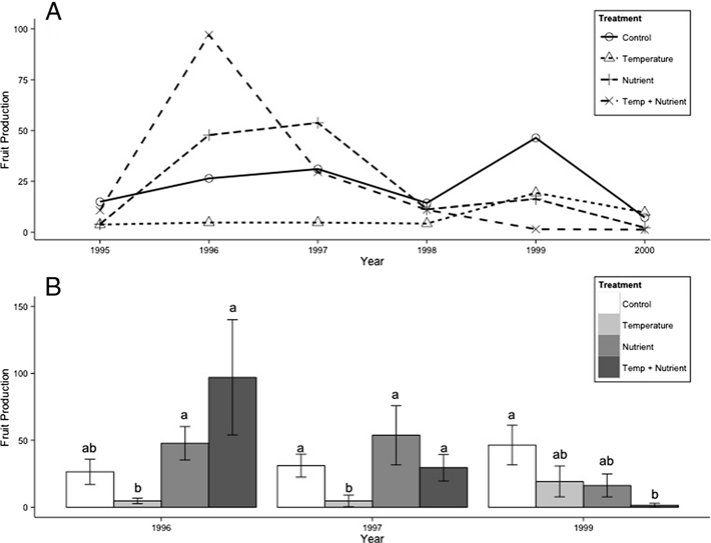
**Response of** ***S. acaulis*** **fruit production to simulated global change. (A)** Yearly fruit production by *S. acaulis* across treatments; in 1995, 1998, and 2000, there were no posthoc differences between fruit production by treatment. **(B)** Differences in mean fruit production by *S. acaulis* plants in different treatments in the three years where temperature or nutrient manipulation had significant effects on flowering. Different letters indicate significant differences between treatments (p < 0.05). In both panels, error bars represent one standard error.

Before the treatments were applied, *S. acaulis* plants produced a mean of 9.65 fruits during the 1995 growing season, with no differences between the warming and control treatments. From 1995 through 2000, the ANOVA model of year, temperature, and nutrient treatments provided a good fit for variation in the fruit data (F = 3.293, d.f. = 23, 96, p < 0.001). Year was the most significant fixed factor in the model, with wide variation in fruits produced between different growing seasons (Figure 4A). Temperature treatment explained significant variation in flowering, as did interactions between nutrient treatment and year, and nutrient treatment and temperature treatment. In 1995, 1998, and 2000, plants across treatments did not produce significantly different numbers of fruits. In 1996, 1997, and 1999, fruits production differed by treatment, and patterns of responses were different in the short term (1996–1997) and medium term (1999) as the manipulations continued (Figure 4B). In 1996 and 1997, plants in the temperature-only treatment produced significantly fewer fruits than those in other treatments; in 1999, it was the temperature plus nutrient manipulation treatment that produced the least fruits, significantly fewer than the control treatment. In fact, the nutrient manipulation seemed to have differing short- and medium-term effects. In 1996 and 1997, plants in the nutrient-only treatment and the combined manipulation treatment produced more fruits than those in the control treatment, though not significantly so. By 1999, the difference between the control and nutrient treatments was still not significant, but mean fruit production was lower in the nutrient treatment, while there was a significant decrease in fruit production in the combined manipulation treatment.

## Discussion

Cushion plants are important facilitator species in alpine ecosystems. Across a seven-year period, the cushion plant *S. acaulis* showed differing short- and medium-term responses to warming and nutrient addition, with the strongest responses often coming in the combined warming and nutrient treatment. For example, over a three-year period, both nutrient addition and the combined warming and nutrient manipulation significantly increased stem elongation, with the combined treatment having a stronger effect. For leaf elongation, only the combined warming and nutrient manipulation had a significant positive effect. Temperature, nutrient, and the combined treatment initially increased cover of *S. acaulis*, but by the seventh year of manipulations, the combined warming and nutrient treatment had a very strong and significant negative effect on cover.

The short term results for vegetative growth indices like stem and leaf elongation are in line with the results obtained from a short-term greenhouse experiment by Hagen and Spomer (1989). Their study on soil temperature and phytohormonal effects on cushion growth of *S. acaulis* showed that both stem and leaf elongation increased significantly with one month laboratory treatment of higher root temperatures and decreased with higher abscisic acid concentrations. We found that *S. acaulis* exhibits considerable phenotypic plasticity in vegetative characters, is highly responsive to changes in its physical environment, and can react quickly to induced changes. The ability to respond quickly to environmental changes can be very beneficial in sub-arctic and arctic areas where harsh conditions are the rule rather than the exception. Thus, when experiencing a warmer than average growing season, *S. acaulis* has the ability to respond by increasing the photosynthetic area and consequently to benefit further from the favorable conditions.

Shaver and Kummerow (1992) suggested that nutrient availability is often a limiting factor for tundra plant growth, which is partly supported by our results as *S. acaulis* was shown to be more responsive to nutrient enhancement than warming. In contrast to our study, Hagen and Spomer’s (1989) study was carried out in a laboratory environment for a period of 30 days, at soil temperatures between 11.5°C to 25°C, and constant air temperatures as high as 21-25°C throughout the experimental period, neither of which can be regarded as normal Arctic or high alpine conditions. Unsurprisingly, while our study showed the same positive short-term reactions in stem and leaf elongation, there are important differences resulting from the different experiment designs. We found that stem and leaf elongation can also be limited by air temperature and nutrient level, and not by soil temperature alone. Since air temperature is more variable than than soil temperature and may thus warm more quickly as a consequence of climate change (Gehrig-Fasel et al. 2008), these results contribute to better understanding of possible consequences of climate change. However, leaf width seems to be more conservative to changes in environmental conditions; this might be due to constraints to rapid changes in morphology.

Cover and biomass increased over the short term in plots experiencing enhanced temperature, nutrient, and the combined warming and nutrient treatments compared to in control plots. Therefore it is likely that the increase in stem and leaf elongation in treatment plots corresponded with a larger increase of above ground growth towards the sides of the cushions (cushion size). However, after five years the initial positive response in cover and biomass had switched to a strong negative response to the combined nutrient and warming treatment, and after seven years the response to the enhanced nutrient level alone had also turned negative (with even further negative impact by the combined nutrient and warming treatment). Favorable conditions for several years in a row are not common in the high alpine and Arctic areas where this species is found. Thus, while being highly responsive to years with favorable conditions, prolonged periods of favorable conditions may have caused *S. acaulis* to be outcompeted within its community. This may also have been the case for *S. oppositifolia* in high Arctic Svalbard where it decreased in response to nutrient addition in a five year study, at the same time the vegetation in the plots experiencing nutrient addition changed from “polar desert” to resemble “bird cliff vegetation” (Robinson et al. 1998).

Morris et al. (2008) suggested that long-lived species would be less sensitive than short-live species to increased variability in climate warming. Cushion plants in high alpine and Arctic areas are generally long-lived and species like *S. acaulis* have been estimated to live for more than 300 years (Morris and Doak 1998), making them prime candidates for this buffering effect. However, our results suggest that a long life span might not be enough to buffer against climate change if environmental changes will induce higher mortality rates, as is indicated by the decrease in cover after the initial increase to the combined nutrient and warming treatment. The buffering capabilities of long-lived species may also be limited to environmental perturbations of a certain scale, and the combination of both warming and nutrient manipulation may have overwhelmed the plants.

A study by Doak and Morris (2010) on four populations across a latitudinal gradient in North America showed that southern populations of *S. acaulis* had lower survival and recruitment, but higher individual growth rates than more northern populations. Furthermore, vital rates such as growth, survival, and fruits per area were shown to increase in moderately warmer years yet declined in the very warmest years, suggesting that a change in climate into warmer conditions or more frequent unusually warm summers may eventually lead to negative impacts (Doak and Morris 2010). Our study gives some experimental support for this latter suggestion if one considers that the environment in the enhanced nutrient and temperature levels may be comparable to a more southern environment with higher temperatures and nutrient levels, caused by higher mineralization rates and microbial activity. While initially experiencing more rapid growth, survival (as indicated by cover) decreased in the medium term for plants experiencing the combination of highest nutrient and temperature regime, just as was shown by Doak and Morris (2010). The greatest negative effects came in the combined temperature and nutrient manipulation, but warming alone also caused initial rapid expansion in cover of *S. acaulis* which then decreased on the medium term, but still remained comparable to its initial value. It is possible that over the longer term, the decline in cover for plants in the warming treatment would continue even further.

We speculate that this decline in cover was due to increased mortality, but the mechanisms remain unclear. Regardless of the cause, the negative effect over the medium term indicates that the plants may have been affected by stress, potentially caused by unusually high investment in vegetative growth (and reproductive effort) for a prolonged period. Stress may also have arisen from other unmeasured factors. Temperature enhancement by OTC’s leads to earlier snowmelt at Latnjajaure (Marion et al. 1997), which may expose plants to potential freezing damage earlier in the growing season; freezing tolerance by *S. acaulis* leaves reduces over the extent of the summer once plants have emerged from the snow, but the degree to which resistance is maintained depends on population and the past frequency of spring and summer freezing events (Junttila and Robberecht 1993). Changes in surface and soil temperature and community productivity may also alter biotic interactions between *S. acaulis* and herbivores, pathogens, and mychorrhizal fungi, all of which could affect survival. Indeed, the importance of biotic interactions and potential decoupling of above- and belowground processes in communities experiencing global change is being increasingly recognized in both the arctic (i.e. Olofsson et al. 2013; Wardle et al. 2013; Rinnan et al. 2009) and across all systems (Van der Putten et al. 2010; Stevbak et al. 2012). Our results indicate that the responses of plants depend on both the abiotic and biotic environment that the population occupies (for example, the core area compared to range margins for the species) and the environmental change that the plants experience.

Even though we found no clear trends in the impact on fruit production, the responses varying between treatments and years, experimental warming has previously been shown to induce earlier flowering, faster fruit development, increased seed production, and a higher seed/ovule ratio in *S. acaulis* (Alatalo and Totland 1997). A previous outcrossing experiment has shown that *S. acaulis* is mainly pollen and not resource limited (Alatalo and Molau 2001). An earlier start to the growing season may be advantageous by enhancing the probability that plants will develop mature seeds before the end of growing season, when the risk of early onset of winter conditions increases at high alpine and Arctic sites. However, fruit production seems to be highly variable based on weather and climate in both the current and previous growing seasons. In this species, flower buds are initiated at the end of the previous growing season, and the more developed the buds are the earlier the plants can begin flowering (Molau et al. 2005).

## Conclusions

If current global change predictions are realistic, with the anticipated warming accompanied by increased nutrient levels due to atmospheric deposits of N and mineralization (Van Cleve et al. 1990), our results suggest that *S. acaulis* may be negatively affected over the longer term. Because *S. acaulis* and other cushion species have important functions as facilitator species, nurse plant (Antonsson et al. 2009) and foundation species across trophic levels (Molenda et al. 2012; Reid and Lortie 2012) in high alpine and Arctic environments, this may in turn cause indirect cascading effects across trophic levels.

## Materials and methods

### Site and study species

The study was carried out in northernmost Sweden at Latnjajaure Field Station (LFS) in the valley of Latnjavagge, 68°21'N, 18°30 T, 1000 m elevation. The valley is covered by snow for most of the year, and the climate is classified as sub-arctic (Polunin 1951; Alatalo and Molau 1995) with cool summers and relatively mild, snow-rich winters (annual minimum ranging from −27.3 to −21.7°C), with a mean annual temperature of −2.0 to −2.7°C (data from 1993–99). Annual precipitation ranges from 605 mm (1996) to 990 mm (1993); the mean for 1990–99 was 808 mm. July is the warmest month with a mean temperature ranging from + 5.2°C (1995) to +8.0°C (1997).

The vegetation in the valley comprises a wide range of communities varying from dry to wet and poor and acidic to base-rich. Even though the geographical situation is subarctic-alpine, the vegetation of the area is representative of the Low Arctic, with *Cassiope tetragona*, *Dryas octopetala*, and *Carex bigelowii* among the dominant species (Molau and Alatalo 1998).

*Silene acaulis (L.)* Jacq. (Caryophyllaceae) is a common cushion-forming plant in alpine and arctic open tundra areas throughout the northern hemisphere and can be found from the Pyrenees and Alps in the south of Europe to the high Arctic. It is a long-lived perennial that forms light green moss-like cushions with pink flowers. The species is polymorphic with reproductive systems and gender frequencies varying between populations (e.g. Alatalo and Molau 1995; Philipp 1997; Alatalo 1997). The *S. acaulis* population at Latnjavagge is part of a rich meadow community, and has a trioecious reproductive system that consists of cushions dominated by female, male, or hermaphrodite flowers. Male flowers have higher pollen viability than hermaphrodites and seed production is mainly limited by pollen availability, not resources (Alatalo and Molau 2001). Mixed cushions with more equal gender expression are rare (Alatalo 1997). The frequency of female plants increases with elevation (Alatalo and Molau 1995).

### Experimental design

The experiment consisted of a factorial combination of two components of environmental manipulations: warming (T) by Open Top Chambers (hereafter OTCs), and nutrient addition (N), with four replicate plots per treatment combination and eight control plots. Thus there were a total of 20 plots measuring 1 x 1 m: 8 control plots and 4 plots for each of the (T, N, and TN) treatments. The plots were selected in the autumn of 1994, and the randomly assigned treatments were implemented in 1995. The OTCs increased warming at the field surface by ca. 3°C (for further information on OTCs and their effect see Marion et al. 1997; Alatalo and Totland 1997). Wet NP addition, 5 g N (as NH_4_NO3 ), and 5 g P (as P2O5), per square meter, diluted in 10 L water, was carried out once a year just after snowmelt in 1995 through 2001.

### Measurements

Growth of stems and leaves were measured following the method of Hagen and Spomer (1989) at the end of the growing seasons 1996, 1997 and 1998. Stem elongation was measured as the distance from the oldest to the youngest green leaf, leaf length and width was measured on the fourth leaf from the apex. The measurements were made with a digital vernier calliper on 10 randomly chosen stems from each treatment plot. The cover of *S. acaulis* in the rich meadow community was measured by using a 1 x 1 m grid-frame with 100 points in each plot and fixed points in each of the corners of the plots; the same grid-frame was used in each measurement to ensure accuracy (Walker 1996). The cover of *S. acaulis* was measured once in mid-August in 1995, 1996, 1997, 1999 and 2001. Biomass was calculated from cover using a scaling factor as in Molau (2010). At the end of each season (late August, 1995–2000), the number of mature capsules (hereafter fruits) was inventoried in all plots. Number of fruits was used as a rough estimate of reproductive success as number of fruits and number of seeds has previously been shown to correlate positively in cushions of *S. acaulis* (Alatalo and Molau 2001).

### Data analyses

Normality and homogeneity of variance were measured using standard diagnostic tests. A three factor ANOVA with temperature, nutrient and year as fixed orthogonal factors was used to analyze potential treatment effects on stem length, leaf length, leaf width, percent cover, biomass, and flowering. When appropriate, posthoc testing was conducted using Tukey HSD tests to detect differences between treatments. Statistics were performed using R (R Core Team 2013).

## Authors’ information

JMA is Senior lecturer at Department of Ecology and Genetics, Uppsala University. CJL is presently master student at Department of Ecology and Genetics, Uppsala University.

## References

[CR1] Alatalo JM (1997). Gender lability in trioecious Silene acaulis (Caryophyllaceae). Nord J Bot.

[CR2] Alatalo J (1998). Climate Change: Impacts on structure and biodiversity of subarctic plant communities.

[CR3] Alatalo JM, Molau U (1995). Effect of altitude on the sex ratio in populations of Silene acaulis (Caryophyllaceae). Nord J Bot.

[CR4] Alatalo JM, Molau U (2001). Pollen viability and limitation of seed production in a population of the circumpolar cushion plant, Silene acaulis (Caryophyllaceae). Nord J Bot.

[CR5] Alatalo JM, Totland Ø (1997). Response to simulated climatic change in an alpine and subarctic pollen‒risk strategist, Silene acaulis. Glob Chang Biol.

[CR6] Antonsson H, Björk RRG, Molau U (2009). Nurse plant effect of the cushion plant Silene acaulis (L.) Jacq. in an alpine environment in the subarctic Scandes, Sweden. Plant Ecol Divers.

[CR7] Arft AM, Walker MDM, Gurevitch J, Alatalo JM, Bret-Harte MS, Dale M, Diemer M, Gugerli F, Henry GHR, Jones MH, Hollister RD, Jónsdóttir IS, Laine K, Lévesque E, Marion GM, Molau U, Mølgaard P, Nordenhäll U, Raszhivin V, Robinson CH, Starr G, Stenström A, Stenström M, Totland Ø, Turner PL, Walker LJ, Webber PJ, Welker JM, Wookey PA (1999). Responses of tundra plants to experimental warming: meta-analysis of the international tundra experiment. Ecol Monogr.

[CR8] Arroyo M, Cavieres L (2003). Positive associations between the cushion plant Azorella monantha (Apiaceae) and alpine plant species in the Chilean Patagonian Andes. Plant Ecol.

[CR9] Bokhorst S, Huiskes A, Convey P, van Bodegom PM, Aerts R (2008). Climate change effects on soil arthropod communities from the Falkland Islands and the Maritime Antarctic. Soil Biol Biochem.

[CR10] Butterfield BJ, Cavieres LA, Callaway RM, Cook BJ, Kikvidze Z, Lortie CJ, Michalet R, Pugnaire FI, Schöb C, Xiao S, Zaitchek B, Anthelme F, Björk RG, Dickinson KJM, Gavilán R, Kanka R, Maalouf J-P, Noroozi J, Parajuli R, Phoenix GK, Reid AM, Ridenour WM, Rixen C, Wipf S, Zhao L, Brooker RW (2013). Alpine cushion plants inhibit the loss of phylogenetic diversity in severe environments. Ecol Lett.

[CR11] Cavieres L, Arroyo M (2002). Nurse effect of Bolax gummifera cushion plants in the alpine vegetation of the Chilean Patagonian Andes. J Veg Sci.

[CR12] Cavieres LA, Sierra-Almeida A (2012). Facilitative interactions do not wane with warming at high elevations in the Andes. Oecologia.

[CR13] Cavieres LA, Brooker RW, Butterfield BJ, Cook BJ, Kikvidze Z, Lortie CJ, Michalet R, Pugnaire FI, Schöb C, Xiao S, Anthelme F, Björk RG, Dickinson KJM, Cranston BH, Gavilán R, Gutiérrez-Girón A, Kanka R, Maalouf J-P, Mark AF, Noroozi J, Parajuli R, Phoenix GK, Reid AM, Ridenour WM, Rixen C, Wipf S, Zhao L, Escudero A, Zaitchik BF, Lingua E, Aschehoug ET, Callaway RM (2014). Facilitative plant interactions and climate simultaneously drive alpine plant diversity. Ecol Lett.

[CR14] Cornelissen JHC, Callaghan TV, Alatalo JM, Michelsen A, Graglia E, Hartley AE, Hik DS, Hobbie SE, Press MC, Robinson CH, Henry GHR, Shaver GR, Phoenix GK, Gwynn Jones D, Jonasson S, Chapin FS, Molau U, Neill C, Lee JA, Melillo JM, Sveinbjornsson B, Aerts R (2001). Global change and arctic ecosystems: is lichen decline a function of increases in vascular plant biomass?. J Ecol.

[CR15] Day TA, Ruhland CT, Strauss SL, Park JH, Krieg ML, Krna MA, Bryant DM (2009). Response of plants and the dominant microarthropod, Cryptopygus antarcticus, to warming and contrasting precipitation regimes in Antarctic tundra. Glob Chang Biol.

[CR16] Doak DF, Morris WF (2010). Demographic compensation and tipping points in climate-induced range shifts. Nature.

[CR17] Dorji T, Totland O, Moe SR, Hopping KA, Pan J, Klein JA (2013). Plant functional traits mediate reproductive phenology and success in response to experimental warming and snow addition in Tibet. Global change biology.

[CR18] Dormann C, Woodin S (2002). Climate change in the Arctic: using plant functional types in a meta‒analysis of field experiments. Funct Ecol.

[CR19] Gehrig-Fasel J, Guisan A, Zimmermann NE (2008). Evaluating thermal treeline indicators based on air and soil temperature using an air-to-soil temperature transfer model. Ecol Modell.

[CR20] Graglia E, Jonasson S, Michelsen A, Schmidt IK, Havström M, Gustavsson L (2001). Effects of environmental perturbations on abundance of subarctic plants after three, seven, and ten years of treatments. Oikos.

[CR21] Hagen SR, Spomer GG (1989). Hormonal regulation of growth form in the arctic-alpine cushion plant, Silene acaulis. Arct Alp Res.

[CR22] Hågvar S, Klanderud K (2009). Effect of simulated environmental change on alpine soil arthropods. Glob Chang Biol.

[CR23] (2007). Climate Change 2007: Impacts.

[CR24] Jägerbrand AK, Alatalo JM, Chrimes D, Molau U (2009). Plant community responses to 5 years of simulated climate change in meadow and heath ecosystems at a subarctic-alpine site. Oecologia.

[CR25] Junttila O, Robberecht R (1993). The influence of season and phenology on freezing tolerance of *Silene acaulis* L., a subarctic and arctic cushion plant of circumpolar distribution. Ann Bot.

[CR26] Klanderud K (2008). Species-specific responses of an alpine plant community under simulated environmental change. J Veg Sci.

[CR27] Le Roux PC, McGeoch MA, Nyakatya MJ, Chown SL (2005). Effects of a short‒term climate change experiment on a sub‒Antarctic keystone plant species. Glob Chang Biol.

[CR28] Makkonen M, Berg MP, van Hal JR, Callaghan TV, Press MC, Aerts R (2011). Traits explain the responses of a sub-arctic Collembola community to climate manipulation. Soil Biol Biochem.

[CR29] Marion G, Henry GHR, Freckrnan DW, Johnstone I, Jones G, Jones MH, Levesque E, Molau U, Molgaard P, Parsons AN, Svoboda J, Virgina RA (1997). Open-top designs for manipulating field temperature in high-latitude ecosystems. Glob Chang Biol.

[CR30] Molau U (1996). Climatic Impacts on Flowering, Growth, and Vigour in an Arctic-Alpine Cushion Plant, Diapensia Lapponica, under Different Snow Cover Regimes. Ecol Bull.

[CR31] Molau U (2010). Long-term impacts of observed and induced climate change on tussock tundra near its southern limit in northern Sweden. Plant Ecol Divers.

[CR32] Molau U, Alatalo J (1998). Responses of subarctic-alpine plant communities to simulated environmental change: biodiversity of bryophytes, lichens, and vascular plants. Ambio.

[CR33] Molau U, Nordenhäll U, Eriksen B (2005). Onset of flowering and climate variability in an alpine landscape: a 10-year study from Swedish Lapland. Am J Bot.

[CR34] Molenda O, Reid A, Lortie CJ (2012). The alpine cushion plant Silene acaulis as foundation species: a bug’s-eye view to facilitation and microclimate. PLoS One.

[CR35] Molina-Montenegro MA, Badano EI, Cavieres LA (2006). Cushion Plants as Microclimatic Shelters for Two Ladybird Beetles Species in Alpine Zone of Central Chile. Arctic, Antarct Alp Res.

[CR36] Morris WF, Doak DF (1998). Life history of the long-lived gynodioecious cushion plant Silene acaulis (Caryophyllaceae), inferred from size-based population projection matrices. Am J Bot.

[CR37] Morris W, Pfister C, Tuljapurkar S (2008). Longevity can buffer plant and animal populations against changing climatic variability. Ecology.

[CR38] Neftel A, Beer J, Oeschger H, Zurcher F, Finkel R (1985). Sulphate and nitrate concentrations in snow from South Greenland 1895–1978. Nature.

[CR39] Olofsson J, te Beest M, Ericson L (2013). Complex biotic interactions drive long-term vegetation dynamics in a subarctic ecosystem. Philos Trans R Soc Lond B Biol Sci.

[CR40] Olsrud M, Carlsson BÅ, Svensson BM, Michelsen A, Melillo JM (2010). Responses of fungal root colonization, plant cover and leaf nutrients to long-term exposure to elevated atmospheric CO2 and warming in a subarctic birch forest. Glob Chang Biol.

[CR41] Philipp M (1997). Genetic diversity, breeding system, and population structure in Silene acaulis (Caryophyllaceae) in west Greenland. Opera Bot.

[CR42] Polunin N (1951). The real Arctic: suggestions for its delimitation, subdivision and characterization. J Ecol.

[CR43] (2013). R: A language and environment for statistical computing.

[CR44] Reid AM, Lortie CJ (2012). Cushion plants are foundation species with positive effects extending to higher trophic levels. Ecosphere.

[CR45] Reid AM, Lamarque LJ, Lortie CJ (2010). A systematic review of the recent ecological literature on cushion plants: champions of plant facilitation. Web Ecol.

[CR46] Rinnan R, Stark S, Tolvanen A (2009). Responses of vegetation and soil microbial communities to warming and simulated herbivory in a subarctic heath. J Ecol.

[CR47] Robinson CH, Wookey PA, Lee JA, Callaghan TV, Press M (1998). Plant community responses to simulated environmental change at a high arctic polar semi-desert. Ecology.

[CR48] Roy J, Albert CH, Ibanez S, Saccone P, Zinger L, Choler P, Clément J-C, Lavergne S, Geremia RA (2013). Microbes on the cliff: alpine cushion plants structure bacterial and fungal communities. Front Microbiol.

[CR49] Shaver GR, Kummerow J, Chapin FI, Jefferies R, Reynolds T, Shaver G, Svoboda J (1992). Phenology, Resource Allocation, and Growth of Arctic Vascular Plants.

[CR50] Stenström M, Gugerli F, Henry GHR (1997). Response of Saxifraga oppositifolia L. to simulated climate change at three contrasting latitudes. Glob Chang Biol.

[CR51] Stevnbak K, Scherber C, Gladbach DJ, Beier C, Mikkelsen TN, Christensen S (2012). Interactions between above- and belowground organisms modified in climate change experiments. Nature Climate Change.

[CR52] Van Cleve K, Oechel W, Horn J (1990). Response of black spruce (Picea mariana) ecosystems to soil-temperature modification in interior Alaska. Can J For Res.

[CR53] Van der Putten WH, Macel M, Visser ME (2010). Predicting species distribution and abundance responses to climate change: why it is essential to include biotic interactions across trophic levels. Philos Trans R Soc Lond B Biol Sci.

[CR54] Walker MD, Molau U, Miolgaard P (1996). Community baseline measurements for ITEX studies. ITEX Man.

[CR55] Wardle DA, Gundale MJ, Jäderlund A, Nilsson M-C (2013). Decoupled long-term effects of nutrient enrichment on aboveground and belowground properties in subalpine tundra. Ecology.

[CR56] Yang Y, Niu Y, Cavieres LA, Sun H (2010). Positive associations between the cushion plant Arenaria polytrichoides (Caryophyllaceae) and other alpine plant species increase with altitude in the Sino-Himalayas. J Veg Sci.

